# Instant Gratification as a Method to Promote Physician Practice Guideline Adherence: A Systematic Review

**DOI:** 10.7759/cureus.9381

**Published:** 2020-07-25

**Authors:** Neha Deo, Emily Johnson, Kaushik Kancharla, John C O'Horo, Rahul Kashyap

**Affiliations:** 1 Medicine, Mayo Clinic Alix School of Medicine, Rochester, USA; 2 Medicine, Oklahoma State University, Stillwater, USA; 3 Medicine, University of Wisconsin, Madison, USA; 4 Infectious Diseases and Pulmonary and Critical Care Medicine, Mayo Clinic, Rochester, USA; 5 Critical Care, Mayo Clinic and Foundation, Rochester, USA

**Keywords:** physician guideline adherence, behavior change wheel, instant gratification, behavioral intervention

## Abstract

Inadequate adherence to best practice guidelines may have a negative impact on the processes of critical care and patient outcomes. Instant gratification has been used to modify human behavior in industries such as gaming, lottery, and social media. We hypothesize that, if properly and purposefully utilized, IG can become a successful tool for encouraging best practice guideline adherence among critical care providers. Four major databases were searched with a medial librarian. Covidence application was used to identify studies pertaining to the instant gratification being used to improve provider adherence with best practice guidelines. A total of 712 studies were identified, and, through duplicates removal, title and abstract screening, and full-text screening, a total of 13 studies were included in the final review. The exclusion criteria used included the following: no provider gratification, wrong focus/intervention, wrong study design, patient-focused intervention, not generalizable, and no conclusion. There is a knowledge gap regarding instant gratification utilization to influence practice guideline adherence among providers. The intervention functions of the Behavior Change Wheel (BCW) were evident, especially ‘persuasion’ and ‘incentivization’, which are most pertinent to our field. The restorative process that promotes positive reinforcement can be a potential solution for alleviating inadequacies in guideline adherence. Examining interventions based on functions of the BCW has shown that an instant gratification process may have the potential in altering critical care providers’ behavior and improving guideline adherence. This review is the first step towards creating smart algorithms to instantly alert providers for their actions compliant with best practices. Developing, testing, and validating the algorithms will be the next several steps.

## Introduction and background

Utilizing physician adherence guidelines can decrease healthcare costs, improve health outcomes for patients, and standardize patient care [1]. However, lack of physician guideline adherence is one of the most prevalent problems in the healthcare system today [2]. Inadequate adherence results in nearly 269 million dollars wasted in treatments that are not beneficial to patients’ health [3]. More importantly, non-compliance with treatment guidelines can also negatively affect a patient's health [2]. However, external barriers such as cost, and internal barriers, lack of knowledge, may make it challenging for healthcare providers to follow guidelines [1]. This may result in a deterioration in the quality of patient care [4].

Recent literature has explored the implementation of behavioral interventions that target patient-centered care [2]. Many of these interventions are based on techniques that have been effective in encouraging providers to follow recommended guidelines. However, it can be difficult to obtain physician buy-in for partaking, especially of those who are not inclined to [5]. Therefore, the development of an effective behavioral intervention requires a rigorous design process that addresses underlying issues that pose a barrier to guideline adherence [6].

One proposed method in developing an effective behavioral intervention is implementing instant gratification. As a phenomenon, it is usually associated with a negative connotation. Instant gratification can be utilized to positively reinforce behaviors that follow guidelines. Electronic health records (EHRs) could be a potential avenue to implement this strategy. The Clinical Decision Support Systems (CDSS) programs improve the quality of care for patients by suggesting additional assessments or test recommendations that the clinician can consider [7]. Recent studies have shown that this tool has been effective in evidence-based practice while also providing an additional resource for knowledge and support [7]. Utilization of CDSS is currently incentivized in the U.S as policy makers believe that the program can cut costs, provide timely reminders, and therefore improve quality of care [4]. 

In order to create a successful intervention that utilizes instant gratification and encourages adherence to guidelines, it is important to utilize the Behavior Change Wheel (BCW). BCW is intended to develop an intervention that analyzes the behavior in question [8]. The underlying hypothesis is that there is a relationship between one's capability (C), opportunity (O), and motivation (M) which explains why a behavior (B) is or is not observed (COM-B) [9]. COM-B could be used to investigate factors that elicit the desired behavior and therefore develop an effective intervention based on clinical context [10].

To date, there has been no behavioral intervention that utilizes instant gratification to improve physician guideline adherence. The purpose of this review is to show that, if properly and purposefully utilized, instant gratification can become a successful tool for encouraging targeted actions and behaviors.

## Review

Methods

**Figure 1:**
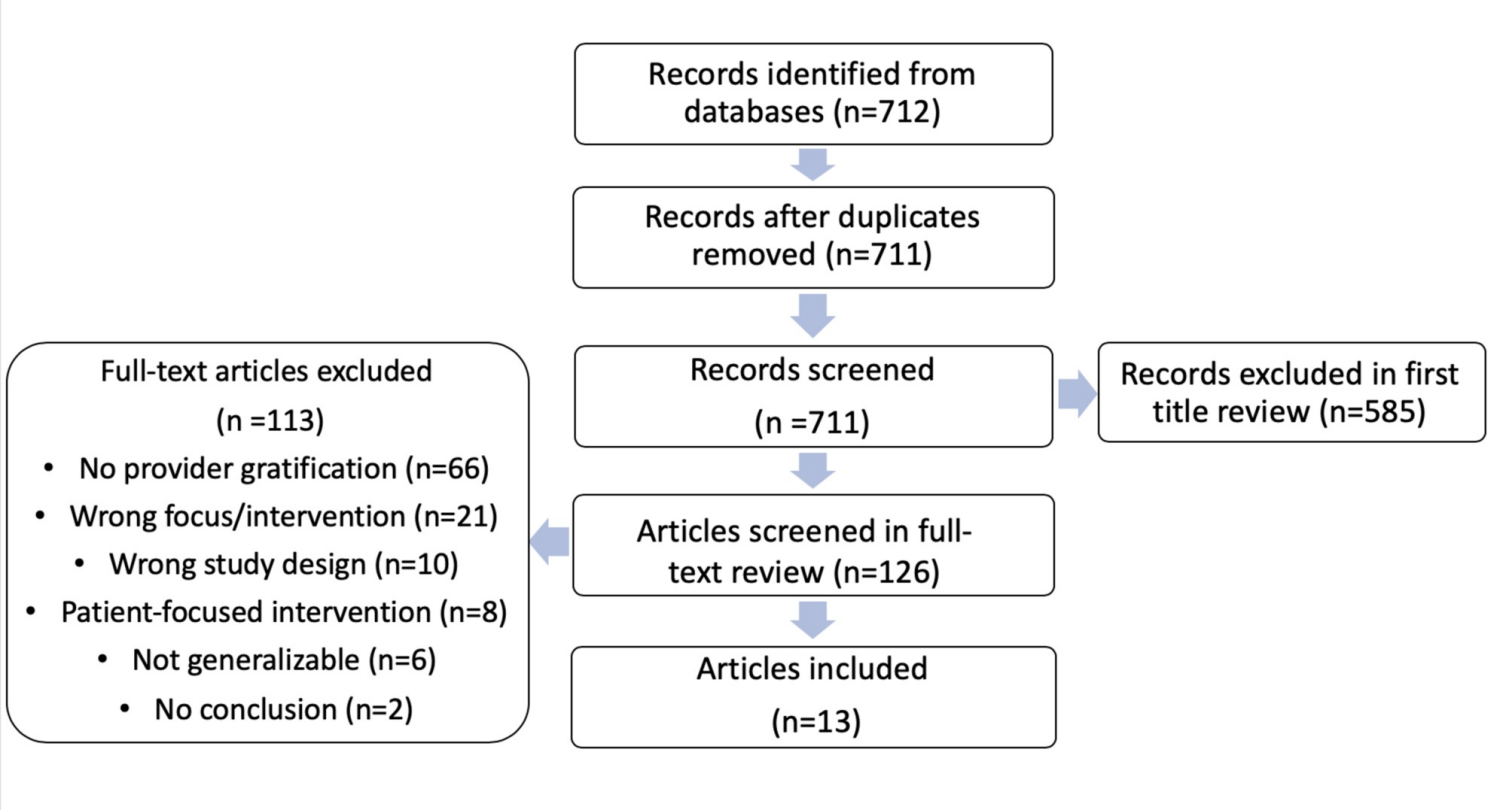
Flowchart of Identification, Screening Exclusion, and Inclusion

We performed a literature review on instant gratification and behavioral interventions in medicine. We provided search terms to a librarian. The librarian searched in four databases (Medline, Embase, CINAHL, Web of Science). The Covidence application was used to identify studies pertaining to using instant gratification to improve physician adherence with practice guidelines. Publications were restricted between January 2009 and December 2018. The primary search term included one of the following: instant gratification, reward, immediate gratification, gratification, short-term gratification, pleasure, impulse, temptation, urge, motivation, or fulfillment. A secondary term, which accompanied the primary term, included one of the following: doctor, nurse, health care providers, healthcare, compliance, treatment compliance, practices, best practices, hospital, dopamine, prefrontal cortex, serotonin, ventral tegmental area, positive reinforcement. A total of 712 records were identified, and, through duplicate screening, title and abstract screening, and full-text screening, 13 studies were included in the final review (Figure 1). The exclusion criteria used included the following: no provider gratification, wrong focus/intervention, wrong study design, patient-focused intervention, not generalizable, and no conclusion.

Results

**Figure 2:**
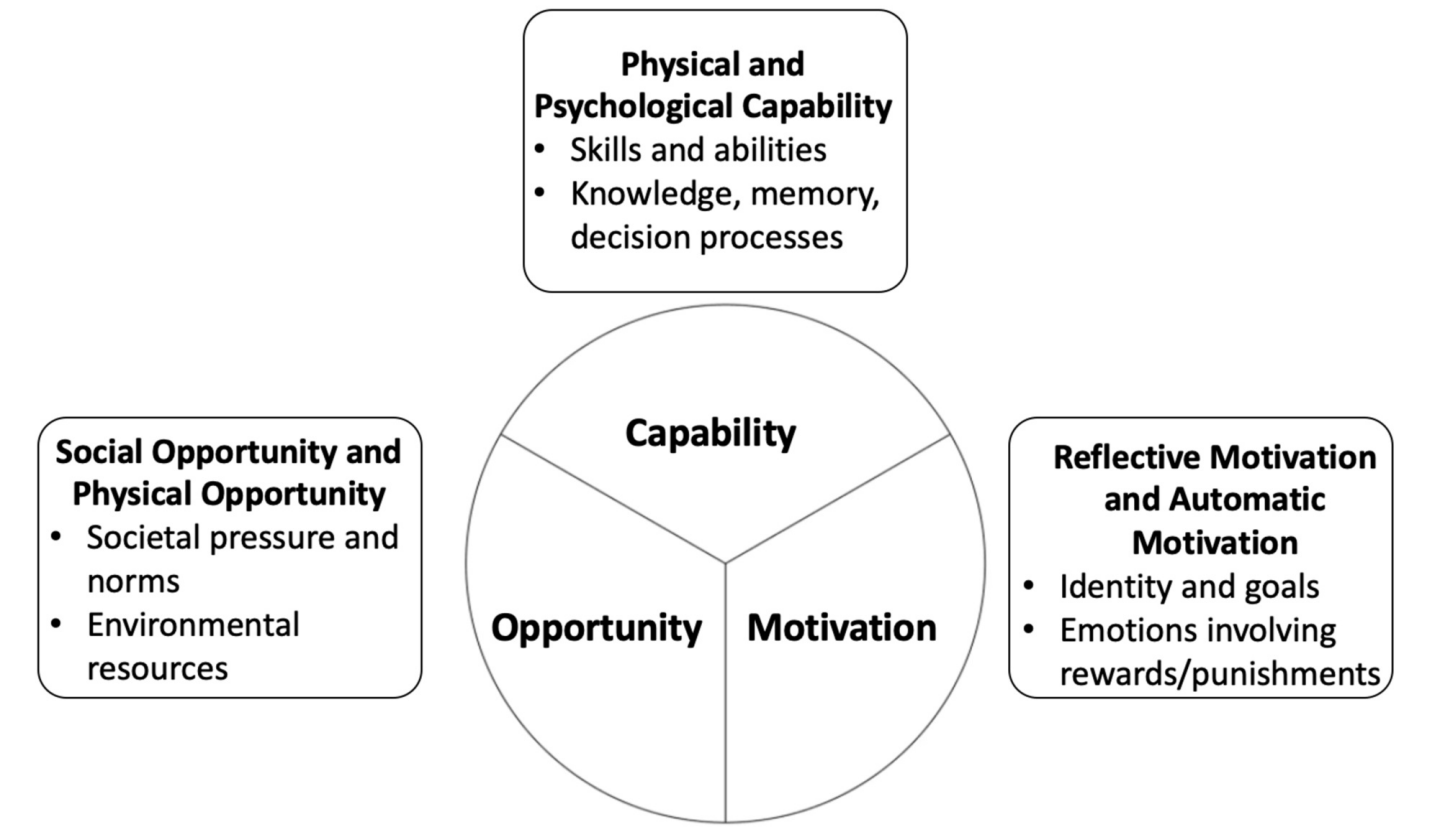
Behavior Change Wheel

The 13 included articles provided a wide variety of information pertaining to the study subject (Table 1). The studies gave information on many of the intervention functions of the behavior change wheel (BCW), especially persuasion and incentivization, which are most relevant to the topic (Figure 2). Intervention development and implementation were both detailed and analyzed, along with potential barriers to interventions.

**Table 1:** Summary of Studies Included in Publication

Author	Study Title, Year	ML Methods	Results	Outcomes/Impact on Healthcare Delivery
Goud et al. [1]	The effect of computerized decision support on barriers to guideline implementation: A qualitative study in outpatient cardiac rehabilitation, 2010	Semi-structured interviews were performed with healthcare professionals who had utilized CARDSS (Cardiac Rehabilitation Decision Support System) for more than 1 year. Professionals from 25 clinics were invited to join the study.	Interviews ranged from 30-60 minutes. Internal barriers, such as lack of familiarity, preference for previous methodologies, and lack of agreement existed. External barriers, such as lack of resources or logistical challenges were a hindrance.	Although utilization of a computerized decision-making support system improved guideline implementation, one must evaluate barriers that may make it difficult for healthcare providers to use this system.
Chauhan et al. [2]	Behavior change interventions and policies influencing primary healthcare professionals’ practice—an overview of reviews, 2017	A systematic review was performed that summarized the effectiveness of different behavioral change interventions.	Clinical decision support systems, multi-faceted teams, and environmental restructuring were found to all have positive effects on adherence to guidelines.	Adherence to guidelines for providers requires a multi-faceted approach that targets effective training, AI systems, and an integrated community with other healthcare workers.
Dixon et al. [4]	Health care providers perceptions of use and influence of clinical decision support reminders: qualitative study following a randomized trial to improve HPV vaccination rates, 2017	Semi-structured interviews with providers aimed to investigate the effectiveness of CDS (Computer-based Clinical Support) programs in pediatric centers that encouraged HPV vaccination.	Providers recalled seeing the notification, however a majority stated that they were not influenced by the notification. However, HPV rates increased during this intervention.	CDS has the potential of influencing provider behaviors, although they may not perceive them to be significant.
Curran et al. [5]	Implementation strategies to promote provider behavior change in emergency departments,	A systematic review was performed that strategies that were implemented to change physician behavior in the emergency department.	Most strategies were multi-faceted, focusing on educational material and reminders.	In order to promote positive behavioral changes in ED physicians, interventions should be intentional and comprehensive.
Creupelandt et al. [6]	Teaching young GPs to cope with psychosocial consultations without prescribing: a durable impact of an e-module on determinants of benzodiazepines prescribing, 2017	GP’s, who were in vocational training, were offered to use an e-module that encouraged psychological support over use of benzodiazepines. GP’s were given an assessment at baseline, 3 months, and 12 months that asked about their perceptions of the program.	GP’s underwent desirable changes related to their self-efficacy and were more likely to adhere to guidelines.	E-modules have the potential to encourage adherence to guidelines, leading to better prescribing practices and less reliance on pharmaceutical drugs.
Durieux et al. [7]	A Clinical Decision Support System for Prevention of Venous Thromboembolism, 2000	Providers used a CDSS (Computer-based decision support system) that provided notifications when seeing patients at risk for venous thromboembolism.	Physicians had an 82.8% compliance to guidelines in control groups and 94.9% in the intervention group. After the intervention, reversion to original compliance was observed.	Integration of CDSS changed provider behavior, however it is clear that ongoing use of the CDSS is integral to continuing those behaviors.
Backman et al. [8]	The development of an intervention to promote adherence to national guidelines for suspected viral encephalitis, 2015	Semi-structured interviews were performed to evaluate barriers to treating patients with viral encephalitis. A suggested intervention was developed. Behavior change techniques were emphasized.	Barriers such as knowledge/skills related to lumbar punctures and organizational resource constraints were highlighted. A core package of suggested interventions were delivered.	This intervention highlighted specific factors that could influence physician behaviors and evaluation when treating patients.
Loft et al. [9]	Strengthening the role and functions of nursing staff in inpatient stroke rehabilitation: developing a complex intervention using the Behaviour Change Wheel, 2017	The behavior change wheel (BCW) was utilized in order to develop an intervention that helps clarify and strengthen the role of nurses in cardiac rehabilitation centers.	An intervention that helped strengthen the functionality of the nurses’ role was developed, and include (but not limited to) interdisciplinary teamwork, training, and educational material.	This intervention was executed in the hospital setting and is currently awaiting results.
Horppu et al. [10]	Application of the Theoretical Domains Framework and the Behavior Change Wheel to Understand Physicians’ Behaviors and Behavior Change in Using Temporary Work Modifications for Return to Work: A Qualitative Study, 2017	The behavior change wheel (BCW) was used as a model to understand physician behaviors when returning to work. Participants participated in focus group interviews relating to temporary work modifications (TWM) when returning to work after an extended period of time.	Promoting desired behaviors were more likely when participants were personally motivated and when resources were not constrained in the workplace. When TWM were applied, physicians were more likely to perform well.	TWM’s are a valuable tool in promoting positive behaviors in physicians that recently have returned to work. In order to positively influence behaviors, systemic changes in the institution should be geared towards support for providers.
Moe-Byrne et al. [11]	Behavior change interventions to promote prescribing of generic drugs: a rapid evidence synthesis and systematic review, 2014	A systematic review was performed to evaluate behavioral interventions that were designed to encourage generic drug prescriptions.	Two studies showed increases in generic prescribing when provided appropriate education. Two others showed promise for electronic subscription. One review highlighted financial incentives. Limited results were found.	Limited evidence is available, however financial incentives and education could potentially influence physician behavior to prescribe generic drugs.
Sinnot et al. [12]	Improving medication management in multimorbidity: development of the Multimorbidity Collaborative Medication Review And Decision Making (MY COMRADE) intervention using the Behavior Change Wheel, 2015	A systematic review was performed, and a qualitative analysis was completed. A focused behavior analysis utilizing the behavior change wheel (BCW) was used to explain why physicians were not actively reviewing medications with patients, and instead following the “status quo”.	Environmental restructuring, incentivization, and enablement were identified as important components to increasing medication review, as well as reviewing medications with another GP.	The BCW can be used to suggest potential solutions for behavioral changes in physicians, particularly with interventions related to multimorbidity.
Pedersen et al. [13]	Can external interventions crowd in intrinsic motivation? A cluster randomised field experiment on mandatory accreditation of general practice in Denmark, 2018	A survey was distributed to participants that asked about the perception of accreditation of general practices in Denmark. Researchers investigated if external interventions could impair physician’s intrinsic motivation.	GP’s viewed the accreditation process as an opportunity for the government to control their practice. Those who viewed it more positively had a higher intrinsic motivation than those who did not.	Some external interventions can motivate physicians towards quality improvement. However, they must see it as a positive factor first.
Ryan et al. [14]	The Intended and Unintended Consequences of Quality Improvement Interventions for Small Practices in a Community-based Electronic Health Record Implementation Project, 2014	A community based EHR program was implemented that focused on clinical decision making. The program either provided feedback, or financial incentives, each time a criterion was met (such as appropriate treatment).	Financial incentives were consistently reported with higher performance. Non- Incentivized measures were associated with lower performance. Technical assistance improved performance measures as well (particularly when incentivized)	Financial incentives and technical support can improve performance outcomes in EHR programs that are designed to support clinical decision making.

We identified 712 records from the literature search and narrowed the results to 13 articles. Of the 13 studies, three studies were reviews of the current literature of behavioral interventions in medicine [2,5,11]. 4 studies utilized the COM-B approach to rigorously evaluate the proposed behavioral intervention for each study [8-10,12]. Another 4 studies tested CDSS programs or modules and measured the frequency of guideline adherence or changes in provider behavior [1,4,6,7]. A total of 7 studies discuss intrinsic or extrinsic factors in healthcare that influence provider motivation [2,8,10-14].

COM-B Approach

A total of 4 studies focused on the COM-B model to enhance their behavioral intervention. Utilizing semi-structured interviews allowed researchers to evaluate the current challenges of providers [8]. Participants are able to express uncertainties related to providing care [8]. Two studies cited that evaluating the clinical environment in terms of resources allowed researchers to create an intervention that addressed these barriers [8,12]. Action planning increased the likelihood that a provider could realistically implement the behavioral intervention in their own clinic [12]. COM-B allowed for researchers to identify the motivations and capabilities of their cohort before designing the intervention [9,10]. Identifying the motivations of individuals was important in enabling providers to elicit the desired behavior [9,10,12].

CDSS Programs 

Four studies utilized CDSS programs or educational modules in the healthcare setting and measured changes in guideline compliance. Providers are likely to notice the notifications that CDSS provides, however they may not perceive it as influencing their behavior - although results may suggest that compliance increased [4]. One study suggested that CDSS increases compliance as it provides clarifications on guidelines for treatment [1]. AI programs increased the frequency of prescribing according to guidelines [6,7]. There was also an increase in self-efficacy in providers when utilizing e-modules [6]. One study noted that if there is a lack of resources available to the provider, online programs will not help compliance [1].

Factors Influencing Motivation

A total of 7 studies explored the influence of intrinsic and extrinsic motivation and changes in behavior. Providing financial incentives led to an increase in the quality of care [11,14]. Addressing external factors, such as lack of experience with procedures, or environmental structuring of healthcare professionals in teams, can influence international motivation and thus performance [2,8,10,12,13]. Organizations that implement audit and feedback programs observe a modest increase in desired behaviors [11].

Discussion

We evaluated the current behavioral interventions that have observed an increase in desired behaviors in healthcare providers. Although there are no studies that have investigated the correlation between instant gratification and guideline compliance, there is plenty of data to suggest that providers can elicit desired behaviors if the right tools are applied. Behavioral interventions utilizing the BCW showed promising results that elicited desirable changes in providers. The success of the BCW comes with its primary goal to evaluate the intervention thoroughly before execution, such as identifying participant motivations and barriers [9,10,12]. Providers that utilized CDSS programs were more likely to adhere to the guidelines and improved prescribing practices. Frequent reminders were a valuable tool for physicians as they provided suggestions for tests or treatment [1]. Individuals who are more motivated to take part in behavioral change techniques are more likely to exhibit the desired behavior [12]. This is fueled by the person’s intrinsic motivation to change. External motivations, such as financial gain, can play a factor, however, it varies from person to person [14].

BCW Interventions

The BCW is a valuable tool that allows researchers to conceptualize targeted behavioral changes, apply the proposed change through an intervention, and then evaluate the results (BCW 1). Proposing an intervention using this method allows researchers to evaluate barriers that would otherwise prevent desirable outcomes [9,10,15]. By utilizing the COM-B method, investigators can develop a strong framework that can focus on a targeted behavior and the outcomes [3,8].

Jackson et al. (2014) encourage the COM-B method for behavioral outcomes related to adherence as it considers external factors that other methods do not consider [3,9]. Behavioral changes in medication adherence can be influenced by a number of external factors such as limited resources and access to healthcare can influence adherence [3,12]. Investigators emphasized the importance of evaluating factors that result in non-compliance through existing literature before executing the intervention [3]. 

Stewart et al. (2019) executed a behavioral intervention which aimed to increase the competency of providers in stroke rehabilitation clinics using the BCW [15]. Participants were found to retain competency in their role after training, were more enthusiastic in their roles, and were more confident in their skills [16]. Long term behavioral changes were observed, as providers were able to recall the learning that was done during the intervention, and felt more satisfied in their role [16]. Investigators stressed that active engagement on behalf of the participants was key to desirable outcomes [16].

Gambling Industry

The gambling industry is a useful comparison for evaluating the potential of instant gratification to elicit behavioral changes. Casinos and lottery games use systems in place to encourage the repetition of gambling behaviors [17]. This is because individuals are hypersensitive to rewards. Mental representations can make gambling more attractive to individuals, and those who have more positive implicit attitudes to gambling are likely to play [17]. By providing spaced intervals in which participants receive satisfaction, addictive behavior is elicited [18].

There is an attractiveness component to uncertain rewards. Intermittent wins and losses are integral to continued play as there is a stronger behavioral response to play when participants cannot predict when rewards come up [17]. Thus, intermittent reward schedules are more resistant to extinction [17]. Players will generally accept more frequent, smaller rewards over larger, longer to wait rewards as it provides a feeling of satisfaction and subjective feelings of pleasure [17,19]. Frequently occurring feelings of pleasure can encourage players to continue gambling, which can soon become a learned association [17]. Some individuals participate in gambling in hopes of monetary gain, however, it is the unpredictability of rewards that motivates one to continue playing [20].

*Social Media* 

Social media usage is largely influenced by gratification, which leads to increases in media usage [21]. There is immediate satisfaction due to content generation and gaining recognition from other users [22], correlating with a positive relationship with social media use [23]. Pleasurable satisfaction can lead to addictive behavior and habit formation. Most users will utilize an online activity to seek gratification, which soon leads to a pattern of conditioned behaviors [24].

Most social media content is created on the basis of motivational factors such as entertainment or information-seeking [22]. If there are greater feelings of satisfaction, individuals are more likely to repeat the same activity [21]. Although specific gratifications are determined by different personality traits [22], once a user develops a habit through gratification, one can expect a self-feedback system in which the user will continue to utilize that medium [23].

Strengths

This study provides valuable information that suggests instant gratification can be utilized to increase guideline adherence in providers. The BCW has the potential to create an intervention that evaluates the needs of the targeted population while also addressing barriers to a specific behavioral response.

Second, the implementation of CDSS programs that suggest recommendations for patient care is highly underutilized, and this paper highlights the effectiveness of these systems. There is a plethora of data to suggest positive outcomes for providers that utilize CDSS programs to improve patient care, and if paired with instant gratification, there is the potential to make these outcomes sustainable in the long term.

Our study also highlights the importance of motivation in behavioral change. If participants are motivated to participate in behavioral interventions, they are more likely to meet the desired outcomes [16]. An important consideration during the pre-intervention phase is to investigate barriers that prevent the desired behavior from being observed. This likely hinders motivation.

Weaknesses

There are several weaknesses that exist in our study. First, there is limited data related to instant gratification in the healthcare setting. Although effective with developed gambling behaviors and with social media usage, these industries heavily rely on developing a habit in the form of an addiction. Many of these addictions develop implicitly. However, we are aiming to create a sustainable behavioral change where providers are intentionally motivated to improve patient care.

Another limitation is that none of our evaluated studies investigated instant gratification. Rather, they evaluated interventions that targeted a certain behavior, and reported outcomes. There is limited information related to instant gratification specifically. Many studies investigate gratification on a broader basis. The effectiveness of instant gratification in behavioral interventions is currently unknown.

## Conclusions

In conclusion, there is the potential for instant gratification to encourage guideline compliance with healthcare providers. The BCW is a valuable tool that evaluates the barriers and motivations of individuals before developing an effective behavioral intervention. Much of the success of a behavioral intervention comes from the participant’s motivation to elicit change. CDSS programs can influence provider behaviors by providing continual reminders to the user. If utilized with instant gratification, it could positively reinforce desired behaviors by providing encouragement to providers. This has the potential to increase guideline compliance if utilized correctly.
